# Enhancing antioxidant capacity and modulating sensory traits by nano‑selenium foliar biofortification on field Leek moss

**DOI:** 10.1016/j.fochms.2025.100341

**Published:** 2025-12-11

**Authors:** Mufan Hu, Hongmei Wang, Cuina Fu, Kouhua Yu, Fengqiong Liu, Qingwei Tan, Yijie Li, Qinyong Dong, Feng Zhang, Shenkui He, Jingcheng Wu, Gengsheng Mo, Yuanhui Xiao, Canping Pan

**Affiliations:** a Guangxi Key Laboratory of Germplasm Innovation and Utilization of Specialty Commercial Crops in North Guangxi, Guangxi Academy of Specialty Crops, Putuo Road 40, Guilin 541004, China; b Department of Applied Chemistry, China Agricultural University, Yuanmingyuan west road 2, Beijing 100193, China; c Agricultural Service Center of Nanbianshan Town, Lingui District, Guilin City, China

**Keywords:** Leek moss, Nano‑selenium, Multi-omics, Sensory traits, Quality, Field test

## Abstract

Nano‑selenium presents a promising biofortification strategy for improving the nutritional and sensory quality of Leek moss (the flower stem of *Allium tuberosum*), yet its molecular basis remains unclear. In a randomized field trial, foliar nano‑selenium application enhanced antioxidant capacity—elevating T-AOC by 45%, SOD/POD/CAT by 20–35% at medium to high concentrations (7.5–10 mg/L), and GSH by 50%—while reducing MDA by 25%. Metabolomic profiling identified 170 differential metabolites, including upregulated flavonoids, phenolic acids, and vitamins (C, B5, B6), alongside reduced sulfurous volatiles such as ACSO and allicin, indicating enhanced nutritional quality and moderated pungency. Transcriptomic analysis revealed 4225 differentially expressed genes, with nano‑selenium specifically activating phenylpropanoid and MAPK signaling pathways, stimulating secondary metabolism and lignin biosynthesis, and repressing CSO biosynthetic genes responsible for pungent flavor. Weighted gene co-expression analysis confirmed nano‑selenium's unique regulatory modules enriched in phenylpropanoid metabolism and reactive oxygen species (ROS) responses. These multi-omics findings demonstrate that nano‑selenium outperforms sodium selenite in promoting antioxidant defense, enhancing beneficial metabolites, and modulating flavor and texture through coordinated transcriptional and metabolic reprogramming. Collectively, this study establishes nano‑selenium foliar biofortification as an effective, sustainable approach for improving both health-promoting and sensory traits of Leek moss.

## Highlights

• Nano-selenium improved the antioxidant capacity of leek moss and increased flavonoids and phenolic acids.
• Reduced ACSO levels and upregulated lignin biosynthesis enhanced sensory traits by decreasing pungency and improving texture.
• Multi-omics confirmed nano-selenium's superior and sustained biofortification efficiency compared with selenite.

## Graphical abstract

**Figure:**
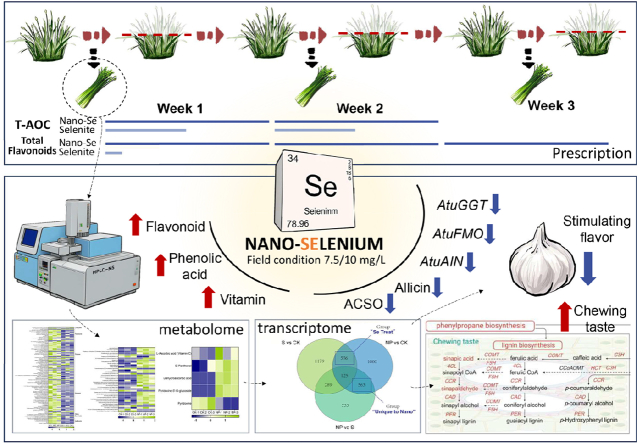

## 1 Introduction

Nanotechnology stands at the forefront of sustainable agriculture, offering innovative strategies to enhance crop growth, improve nutritional quality, and strengthen stress tolerance (Kah, Tufenkji, & White, 2019; Sun et al., 2022; Zaman, Ayaz, & Park, 2025). However, its application toward enhancing the edible quality and sensory attributes of crops remains relatively underexplored, particularly in Allium species such as the Chinese chive. In vegetable crops, yield, quality, and palatability collectively define market value, therefore, assessing how exogenous inputs—such as nanomaterials—influence these characteristics is essential for optimizing both crop performance and consumer acceptance.

Chinese chive (*Allium tuberosum* Rottler ex Sprengel) is a widely cultivated leafy vegetable in East, Southeast, and South Asia, where it is commonly used both as a vegetable and as a pungent aromatic herb (Kothari et al., 2020). It is valued for its rich nutritional composition, including moderate levels of protein and dietary fiber (approx. 3.1 g and 2.6 g per 100 g fresh leaves, respectively), along with essential vitamins (e.g., vitamin C, carotenoids) and minerals (e.g., K, Ca, Fe, Se) that contribute to its antioxidant potential (Gavhane et al., 2024). Like other Allium species, accumulates a variety of bioactive compounds, such as organosulfur volatiles (e.g., thiosulfinates), flavonoids, and steroidal saponins, which collectively confer strong antimicrobial and antioxidant activities. In addition to its leaves, the edible flowering stalk—known as Leek moss—is also highly esteemed, particularly in Chinese cuisine. This structure differs from the leaf in morphology, flavor, and culinary function. Leek moss exhibits a sharper, garlic-like flavor than the leaf but remains milder than garlic cloves. It is especially appreciated for its crisp, fibrous texture and the distinctive “snap” it produces when bitten. By contrast, the broad, flat leaves provide a softer, more tender mouthfeel and are typically eaten fresh or lightly cooked, similar to scallions. Despite their comparable nutritional and phytochemical profiles, the stalk's firmer texture and enhanced sulfurous aroma distinguish it in culinary applications, particularly in stir-frying and pickling. Sensory quality—especially flavor and texture—plays a pivotal role in consumer perception of vegetable crops, and in Chinese chive these attributes are tightly linked to metabolism. Pungency arises from sulfur-containing metabolites such as S-alk(*en*)yl-L-cysteine sulfoxides, which are hydrolyzed by alliinase into volatile thiosulfinates (e.g., allicin) upon tissue disruption (Wang, Zhang, Li, Chai, & Xie, 2025). These compounds impart the distinctive aroma and sharp flavor characteristic of the Allium genus (Dai, Azi, Alnadari, & Yu, 2025). Textural properties, on the other hand, are primarily governed by cell wall architecture, particularly lignin accumulation. Lignin—a phenolic polymer derived from the phenylpropanoid pathway—reinforces cell walls, increasing tissue rigidity and mechanical strength (Polo et al., 2020). While greater lignin deposition enhances firmness, it can also reduce chewability. Thus, the characteristic flavor and texture of Leek moss are closely associated with its sulfur metabolism and lignification processes, underscoring the biochemical foundation of its unique edible traits.

Selenium (Se), an essential micronutrient, has been widely recognized for its ability to stimulate plant growth and enhance antioxidant enzyme activities, thereby strengthening plant tolerance to environmental stress (Huang, Tan, Wang, & Zhou, 2024). Functioning as the catalytic center of glutathione peroxidase, Se plays a pivotal role in activating the ascorbate–glutathione (AsA–GSH) cycle by utilizing glutathione (GSH) as a substrate (Jia et al., 2023). However, most previous research on Se fertilization has relied on sodium selenite or selenate, which possess relatively low bioavailability and higher phytotoxicity due to their chemical characteristics (Li, An, Wu, Li, & Pan, 2020). Nanotechnology provides a promising alternative, as it can increase the bioavailability and catalytic efficiency of Se while simultaneously minimizing toxicity (Gao et al., 2023; Ye, Sun, Cai, & Jiang, 2024). Nano‑selenium—a zero-valent, nanoscale form of Se synthesized through nanotechnology—has exhibited a markedly greater ability to activate antioxidant enzymes and modulate reactive oxygen species (ROS) metabolism, thereby promoting plant growth and enhancing chloroplast functionality (Abbas, Anjum, Fatma, & Ahmad, 2025). Its efficacy in improving crop quality and mitigating both biotic and abiotic stresses has consequently drawn increasing scientific attention. Recent studies in pak choi (Wang et al., 2023), cucumber (Jia et al., 2024), and cherry tomato (Cheng et al., 2022) have demonstrated that nano‑selenium enhances plant resilience and quality by stimulating photosynthetic performance, elevating soluble metabolite accumulation and nutrient content, maintaining ROS homeostasis, and stabilizing cellular osmotic balance. However, the physiological responses to nano‑selenium are often species-specific, and beyond enhancing nutritional quality and stress resistance, nano‑selenium may also modulate sensory traits such as flavor and texture. To date, no direct studies have investigated the use of nanomaterials—including nano‑selenium—in Chinese chive.

Building on previous reports that nano‑selenium enhances vegetable quality and stress tolerance, we hypothesized that foliar nano‑selenium application could enhance the resilience, nutritional value, and sensory characteristics of leek moss. In this study, nano‑selenium was applied through foliar spraying, and its biofortification effects were systematically evaluated under field conditions. Agronomic traits, along with physiological and biochemical parameters, were monitored across three consecutive harvests to assess both the impact and persistence of nano‑selenium treatment. Furthermore, integrated multi-omics analyses were performed to elucidate the molecular and metabolic mechanisms underlying nano‑selenium–induced changes in leek moss quality and edibility. Collectively, this study provides transcriptomic, metabolomic, and physiological evidence demonstrating the potential of nano‑selenium as an effective biofortification strategy for improving leek moss production and quality.

## 2 Materials and methods

### 2.1 Synthesis of Nano‑selenium

A 1% chitosan solution was prepared as a premix, followed by the slow addition of 20 mL of 20 mM sodium selenite solution. The mixture was stirred continuously at 500 rpm and 25 °C to obtain a nano-dispersed selenite colloid. Subsequently, 4 mL of 1% ascorbic acid solution was added dropwise under the same stirring conditions for 3 h. The reaction was considered complete when the solution turned transparent red, indicating successful synthesis of nano‑selenium. The physicochemical stability and morphology of the synthesized Nano‑selenium were confirmed by transmission electron microscopy (TEM) prior to foliar application, as previously described in our celery-based application study (Kang et al., 2023; Li et al., 2020).

### 2.2 Plant culture

The field trial was conducted from April to May 2024 in Nanxin Village, Nanbian Mountain Town, Lingui District, Guilin City. The soil type was sandy loam. A ridge cultivation system was used, with ridges 120 cm wide, 30 cm high, and furrows 40 cm wide. The tested Leek moss cultivar was ‘Nianhua 13’, transplanted on March 6, 2023, using double plants per hole with a spacing of 25 cm × 30 cm. Exogenous selenium was applied as nano‑selenium and sodium selenite at concentrations of 2.5, 5.0, 7.5, 10 and 15 mg/L, along with a water control, resulting in seven treatments. Foliar spraying was performed every seven days, for three consecutive applications, using the standard field practice of spraying until the leaf and stalk surfaces were evenly moistened. The experiment followed a randomized block design with a plot size of 25 m^2^ and three replicates per treatment under conventional cultivation practices. Spraying was carried out on April 23, April 29 (advanced due to forecasted heavy rain on April 30), and May 6. Sampling was conducted on May 13, May 20, and May 27. Thirty Leek moss stalks were randomly collected per plot to measure stalk height, diameter, and single stalk weight. Mid-sections (100 g) were flash-frozen in liquid nitrogen and stored at −80 °C. Yield was recorded daily from May 20 to June 3 for each plot.

### 2.3 Growth, physiological, and biochemical parameters of Chinese chives

Commercial assay kits (Nanjing Jiancheng Bioengineering Institute, Nanjing, China) were used to quantify soluble sugars (anthrone‑sulfuric acid method), soluble proteins (Coomassie brilliant blue method), total flavonoids (aluminum chloride colorimetric method), and total phenolics (Folin–Ciocalteu method). Chlorophyll a and b contents were determined spectrophotometrically. Briefly, samples were washed, air-dried, and 0.5 g of tissue was weighed and finely chopped. The tissue was ground with 2 mL of 96% ethanol, a small amount of calcium carbonate, and quartz sand until fully homogenized and decolorized. After standing in the dark for 5 min, the homogenate was filtered and the filtrate was adjusted to 25 mL. Absorbance was measured at 663 nm for chlorophyll *a* and 645 nm for chlorophyll *b*.

### 2.4 Determination of total and inorganic selenium contents

Total selenium in leek moss (CK, 7.5 mg/L nano selenium and inorganic selenium) tissues was determined following an accredited elemental analysis procedure. Briefly, oven-dried and homogenized samples (1–5 g fresh weight) were digested in Teflon vessels with 5 mL concentrated HNO₃ using a stepwise temperature program (80 °C for 2 h, 120 °C for 2 h, and 160 °C for 4 h). After evaporation to near dryness and dilution to 25 mL with 1% HNO₃, the digests were quantified by inductively coupled plasma mass spectrometry (ICP-MS) using internal standard calibration. Reagent blanks were included in each batch and used for background correction. Inorganic selenium was extracted by 6 mol L^−1^ HCl under ultrasonic treatment (45 min) followed by a boiling water bath (30 min). The filtrate was evaporated, redissolved in HCl and reduced to Se (IV). Selenium hydride was generated with NaBH₄ and quantified by atomic fluorescence spectrometry (AFS) using external standard curves. All analyses included procedural blanks. Organic selenium content was calculated as the difference between total and inorganic selenium.

### 2.5 Measurement of antioxidant substances and enzyme activity

Total antioxidant capacity (T-AOC), superoxide dismutase (SOD), peroxidase (POD), catalase (CAT), glutathione peroxidase (GSH-Px), glutathione (GSH), and malondialdehyde (MDA) were quantified using commercial assay kits (Nanjing Jiancheng Bioengineering Institute, Nanjing, China). TBARS (MDA equivalents) was measured as an indicator of lipid peroxidation and oxidative stress. Antioxidant enzymes, including SOD (EC 1.15.1.1), POD (EC 1.11.1.7), and CAT (EC 1.11.1.6), were detected using corresponding assay kits. Briefly, 100 mg of Leek moss powder was extracted with 1 mL phosphate buffer (pH 7.0) or manufacturer-supplied extraction reagent. Homogenization was performed at 8000 rpm for 10 min at 4 °C. Antioxidant parameters were determined using the supernatant following kit instructions at specific wavelengths.

### 2.6 Widely targeted metabolomic analysis

Samples were freeze-dried (Scientz-100F) for 63 h and ground into powder. Then, 50 mg of powder was extracted with 1200 μL of pre-chilled (−20 °C) 70% methanol containing internal standards (prepared by diluting 1 mg/mL stock solution of standard to 250 μg/mL with 70% methanol). Samples were vortexed every 30 min for 30 s (6 times total), centrifuged at 12,000 rpm for 3 min, and the supernatant was filtered through a 0.22 μm membrane for LC-MS/MS analysis.

Metabolomic profiling was performed using ultra-performance liquid chromatography (UPLC, ExionLC™ AD) coupled with tandem mass spectrometry (MS/MS). Chromatographic separation was achieved using an Agilent SB-C18 column (1.8 μm, 2.1 mm × 100 mm). The mobile phase consisted of solvent A (ultrapure water with 0.1% formic acid) and solvent B (acetonitrile with 0.1% formic acid). The elution gradient was: 0.00 min, 5% B; linear increase to 95% B over 9.00 min; held at 95% for 1 min; decreased to 5% from 10.00 to 11.10 min; re-equilibrated at 5% B until 14 min. Flow rate: 0.35 mL/min; column temperature: 40 °C; injection volume: 2 μL. Mass spectrometry was conducted using an electrospray ionization (ESI) source at 500 °C. Ion spray voltages were set to +5500 V (positive mode) and −4500 V (negative mode). Gas settings were: GS1 at 50 psi, GS2 at 60 psi, curtain gas at 25 psi. Collision-induced dissociation (CID) was set to high. Data were acquired in MRM (multiple reaction monitoring) mode using QQQ scanning, with nitrogen as the collision gas at medium pressure. Declustering potential (DP) and collision energy (CE) were optimized for each MRM transition, which were monitored in time-scheduled acquisition windows based on metabolite retention times.

### 2.7 RNA‑seleniumq analysis and gene identification

Total RNA was extracted from 80 mg of Leek moss tissue using the CTAB-PBIOZOL method followed by ethanol precipitation. RNA quality and quantity were assessed using the Qubit 4.0 Fluorometer/MD microplate reader and Qsep400 Bioanalyzer. For transcriptome analysis, RNA from 80 mg of young leaves was similarly purified and evaluated using NanoDrop and Agilent 2100. After RNA-seq library construction, quality control was performed on the Agilent 2100 Bioanalyzer, and sequencing was conducted on the Illumina NovaSeq 6000 platform. Each sample yielded >6 Gb of clean data, with Q30 scores ≥93%. Clean reads were mapped to the reference genome of *Camellia sinensis* var. shucha. Gene expression levels were normalized as FPKM (Fragments Per Kilobase of transcript per Million mapped reads). Gene annotation and functional enrichment were performed using Gene Ontology (GO) and Kyoto Encyclopedia of Genes and Genomes (KEGG) databases. Differentially expressed genes (DEGs) were identified with thresholds of |log₂ fold change| ≥ 1.0 and FDR < 0.05.

### 2.8 Data analysis

Graphs were generated using GraphPad software. Statistical analyses were performed with SPSS (IBM SPSS, version 27.0). Independent sample *t*-tests, one-way ANOVA, and two-way repeated measures ANOVA were conducted with a significance threshold of *p* < 0.05. Principal component analysis (PCA), partial least squares discriminant analysis (PLS-DA), orthogonal PLS-DA (OPLS-DA), volcano plots, and heatmaps were carried out using RStudio. Metabolic pathway analysis was based on the KEGG database.

## 3 Results

### 3.1 Effects of sodium selenite and nano‑selenium on physiological and biochemical parameters in Chinese chive moss

**Fig. 1:**
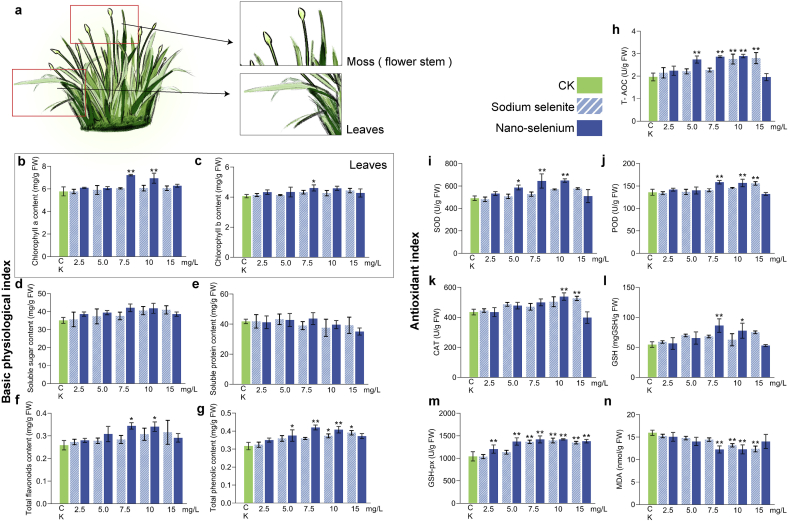
**Physiological, biochemical, and antioxidant parameters.** (a) Diagram of Chinese chive tissue structure; (b) Chlorophyll a content; (c) Chlorophyll b content; (d) Soluble sugar content; (e) Soluble protein content; (f) Total flavonoid content; (g) Total phenolic content; (h) Total antioxidant capacity (T-AOC); (i) Superoxide dismutase (SOD) activity; (j) Peroxidase (POD) activity; (k) Catalase (CAT) activity; (l) Glutathione (GSH) content; (m) Glutathione peroxidase (GSH-Px) activity; (n) Malondialdehyde (MDA) content. Selenium concentration is expressed based on elemental Se content.

To optimize field application rates of inorganic selenium and nano‑selenium in Leek moss and assess their effects on physiological and biochemical parameters, a completely randomized block design field trial was conducted using foliar applications of sodium selenite and nano‑selenium. Given the short growth period of Leek moss, with only ∼15 days from scape emergence to harvest, harvesting commenced in the fourth week, following three weeks of Se treatment. As the scape is the reproductive organ and leaves are the main photosynthetic organs (Fig. 1a), photosynthetic assessments were conducted on the leaves. Chlorophyll a, a core component of PSII, plays a central role in oxygenic photosynthesis. Compared with the control (CK), 7.5 mg/L and 10 mg/L nano‑selenium significantly increased chlorophyll *a* content by 24.9% and 20.1%, respectively (Fig. 1b). Chlorophyll b also increased significantly (13.4%) under 7.5 mg/L nano‑selenium treatment (Fig. 1c). These findings align with previous reports that Se enhances photosynthesis (García-Locascio, Valenzuela, & Cervantes-Avilés, 2024). In contrast, sodium selenite showed no significant effect on either chlorophyll *a* or b, indicating that the applied concentrations may have been below the effective threshold. Photosynthetic pigments are critical for light harvesting, directly influencing the dark reaction and soluble sugar synthesis. Soluble sugars enhance sweetness and reduce astringency in Leek moss. Although nano‑selenium significantly increased leaf pigment levels, its effect on soluble sugar content was not statistically significant within the 0–10 mg/L range, despite a slight increasing tendency observed in the data (Fig. 1d). Soluble protein content remained unchanged across treatments (Fig. 1e). Flavonoids are key bioactive components in Leek moss. Selenium has been shown to promote secondary metabolism, particularly enhancing antioxidant-related compounds like flavonoids and phenolic acids. Compared with CK, nano‑selenium at 7.5 mg/L and 10 mg/L significantly increased total flavonoid content by 32.9% and 31.6%, respectively (Fig. 1f). Total phenolic content was significantly elevated under nano‑selenium at 5.0 mg/L (12.9%), 7.5 mg/L (26.0%), and 10 mg/L (24.8%), and under sodium selenite at 10 mg/L (12.5%) and 15 mg/L (15.6%) (Fig. 1g). These results demonstrate the superior efficacy of nano‑selenium over sodium selenite at equivalent concentrations. The optimal nano‑selenium concentration for enhancing secondary metabolism lies between 7.5 and 10 mg/L, while sodium selenite requires >15 mg/L for comparable effects.

Studies have shown that selenium enhances plant stress and disease resistance by reducing malondialdehyde (MDA) levels and activating the GSH-AsA antioxidant cycle and antioxidant enzymes(Ghanbari, Bag-Nazari, & Azizi, 2023; Tavanti et al., 2021; Wang et al., 2023). In this study, exogenous Se significantly improved the total antioxidant capacity (T-AOC) of Leek moss (Fig. 1h). Nano‑selenium at 5, 7.5, and 10 mg/L increased T-AOC by 39.5%, 45.9%, and 47.4%, respectively. Sodium selenite at 10 and 15 mg/L raised T-AOC by 40.5% and 42.7%. Beyond secondary metabolites, enzymatic antioxidant activity plays a key role in T-AOC. Superoxide dismutase (SOD) activity significantly increased by 19.7%, 34.8%, and 32.5% under 5, 7.5, and 10 mg/L nano‑selenium, and by 16.2% under 15 mg/L sodium selenite (Fig. 1i). Peroxidase (POD) activity was enhanced by 16.9%, 15.6%, and 14.9% under 7.5 and 10 mg/L nano‑selenium and 15 mg/L sodium selenite, respectively (Fig. 1j). Catalase (CAT) activity increased by 23.7% with 10 mg/L nano‑selenium and 21.0% with 15 mg/L sodium selenite (Fig. 1k). As a functional component of glutathione (GSH), Se is integral to the AsA-GSH antioxidant cycle. Nano‑selenium at 7.5 and 10 mg/L significantly boosted GSH levels by 58.1% and 42.3%, respectively (Fig. 1l). All nano‑selenium treatments significantly enhanced GSH-Px activity, peaking at 36.1% under 7.5 and 10 mg/L (Fig. 1m). In contrast, sodium selenite only showed significant effects at 7.5–15 mg/L, with a peak increase of 33.7% at 10 mg/L. Collectively, exogenous Se markedly improved antioxidant capacity in Leek moss. Given that SOD, POD, GSH, and GSH-Px are core components of the AsA-GSH cycle, results strongly suggest Se application activated this system. Consequently, appropriate Se concentrations significantly reduced MDA levels (Fig. 1n), indicating enhanced cellular resistance and protection against oxidative stress.

### 3.2 The application of nano‑selenium maintains efficacy in continuously harvested Chinese chive moss

**Fig. 2:**
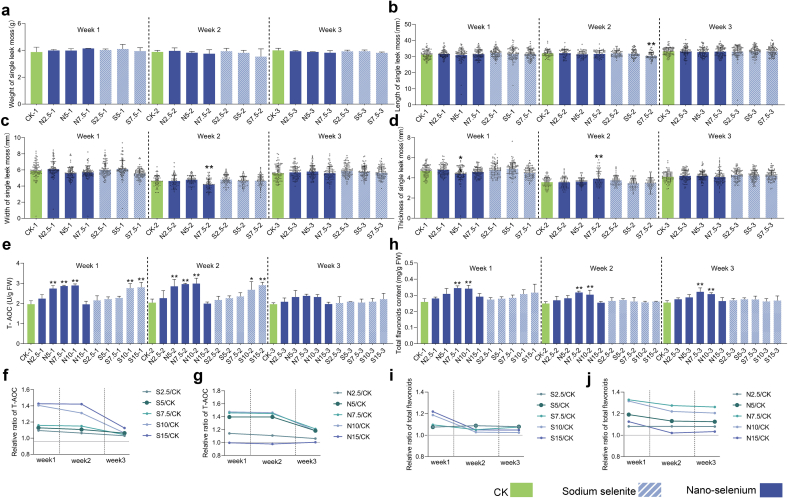
**Evaluation of agronomic traits and temporal efficacy of nano-selenium application.** (a) Average single stalk weight of *Allium tuberosum*; (b) Average stalk length; (c) Average stalk width; (d) Average stalk thickness; (e) T-AOC of *A. tuberosum* over three consecutive weekly harvests; (f) Temporal changes in T-AOC under sodium selenite (S) treatment; (g) Temporal changes in T-AOC under nano-selenium (N) treatment; (h) Total flavonoid content over three consecutive harvests; (i) Temporal dynamics of flavonoid content under S treatment; (j) Temporal dynamics of flavonoid content under N treatment. “S” and “N” indicate sodium selenite and nano-selenium treatments, respectively. For example, “S2.5–1” denotes samples treated with 2.5 mg/L sodium selenite and collected during the first week of harvest. The determination of agronomic traits used 50 Leek moss as biological replicates.

In practical production, the persistence of exogenous interventions is a key parameter for evaluating applications in continuously harvested crops. As shown in Section 3.1, the optimal concentration of nano‑selenium falls between 7.5 and 10 mg/L. Higher concentrations (>10 mg/L) negatively affected plant performance. Considering material and environmental costs, 7.5 mg/L was selected as the optimal application rate, and concentrations ranging from 0 to 7.5 mg/L were used for agronomic assessment. After foliar application, Leek moss were harvested weekly for three weeks (Week 1–3). Average weight, length, width, and thickness per moss were recorded to assess phenotypic and yield responses. Results showed no significant impact of Se application on overall yield (Fig. 2a–d). Although some treatments caused significant changes in length, width, or thickness during Week 1 and Week 2, no consistent trend emerged. Analysis of average moss weight revealed a slight increase with increasing nano‑selenium concentration in Week 1; 7.5 mg/L nano‑selenium led to a 6.9% yield increase. However, in Weeks 2 and 3, moss weight declined with increasing Se concentration—a trend also observed in the sodium selenite groups. This pattern may reflect a shift in growth strategy. Under stress, plants often suppress vegetative growth in favor of reproductive development (e.g., early flowering) to complete their life cycle (Wada & Takeno, 2010). Selenium enhanced the antioxidant capacity of the plants, alleviating stress and promoting vegetative over reproductive growth. Consequently, fewer flower stalks were formed in later stages (Week 2 and 3), leading to reduced yields. In contrast, Leek mosses harvested in Week 1, having developed under favorable conditions with sufficient time, showed a positive yield response.

Based on the significant improvements in antioxidant capacity and secondary metabolism following selenium application, T-AOC and total flavonoids were selected as indicative markers to evaluate the persistence of these effects. Results showed that T-AOC levels in Leek moss were significantly elevated in Week 1 and Week 2 after optimal selenium treatment, but declined below significance in Week 3 (Fig. 2e). Fold change analysis relative to CK over time (Fig. 2f, g) revealed that both sodium selenite and nano‑selenium effectively enhanced antioxidant capacity for up to two weeks, with nano‑selenium at 10 mg/L showing a slightly longer duration of enhancement. Regarding secondary metabolism, total flavonoid levels showed consistent significant increases relative to the control across three harvests (Fig. 2h). In contrast, the effects of sodium selenite declined sharply from Week 1 to Week 2 (Fig. 2i). Notably, lower concentrations of nano‑selenium produced a moderate but consistent enhancement across three harvests, with slower attenuation rates. (Fig. 2j). These findings indicate that nano‑selenium provides more persistent enhancement of antioxidant capacity and secondary metabolism than sodium selenite, particularly at lower concentrations.

**Supplementary Figure S1:**
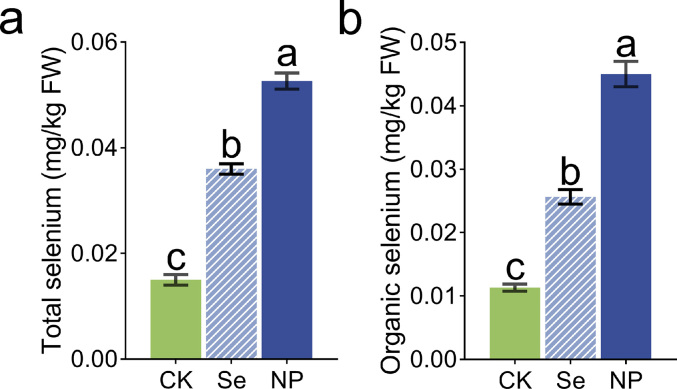
**Determination of total selenium (a) and organic selenium (b) contents in leek moss harvested in Week 1 under foliar application of 7.5 mg**/**L sodium selenite and nano-selenium.** Different lowercase letters indicate significant differences among treatments according to one-way ANOVA followed by post-hoc multiple comparison (*p* < 0.05).

To further ensure food safety and assess selenium biofortification levels, total and organic selenium contents were quantified in leek moss harvested during the first week after foliar application (7.5 mg/L sodium selenite or nano selenium, Fig. S1). Both selenium forms markedly increased tissue Se accumulation compared with the non-treated control. In the absence of external Se, total and organic selenium contents were 0.014 and 0.011 mg kg^−1^, respectively. Application of 7.5 mg/L sodium selenite increased these values to 0.036 and 0.025 mg kg^−1^, whereas nano‑selenium application led to a higher accumulation of 0.053 and 0.045 mg kg^−1^, respectively. According to the classification of selenium-enriched agricultural products in standard NY/T 3115–2023, both treatments enabled leek moss to reach the “high‑selenium” grade (0.30–1.00 mg kg^−1^). Importantly, the observed selenium levels were within the range considered safe for consumption, indicating that nano‑selenium foliar biofortification can effectively enhance Se content without compromising food safety.

### 3.3 Metabolomics analysis reveals enhanced secondary metabolism and nutritional value in Leek moss by nano‑selenium

**Fig. 3:**
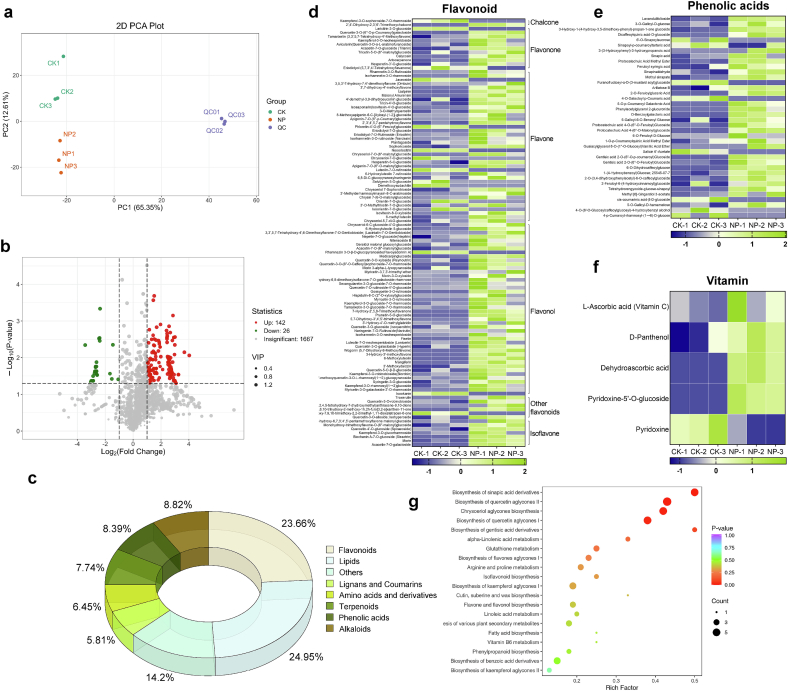
**Metabolomic analysis.** (a) Principal component analysis (PCA), CK represents the control group, NP represents the nano-selenium treatment group (7.5 mg/L), and QC represents the quality control samples; (b) Volcano plot of differential metabolites; (c) Pie chart of differential metabolite classifications; (d) Heatmap of significantly altered flavonoid compounds; (e) Heatmap of significantly altered phenolic acids; (f) Heatmap of significantly altered vitamins; (g) KEGG pathway enrichment analysis. In the heatmaps, color intensity reflects the normalized relative abundance of metabolites, expressed as *Z*-scores.

Based on the previously determined optimal field concentration of nano‑selenium for Leek moss, a broad-target metabolomic analysis was conducted for the control (CK) and 7.5 mg/L nano‑selenium group (NP). Principal component analysis (Fig. 3a) showed clear separation among CK, NP, and QC samples. A total of 170 differential accumulated metabolites (DAMs) were identified between NP and CK, including 142 upregulated and 28 downregulated compounds (Fig. 3b). These DAMs were mainly flavonoids (23.66%), lipids (24.95%), lignans and coumarins (5.81%), amino acids and derivatives (6.45%), terpenoids (7.74%), phenolic acids (8.39%), and alkaloids (8.82%).

Flavonoids and phenolic acids, as major dietary polyphenols, exert complementary health benefits via diverse biochemical mechanisms. Flavonoids are potent antioxidants with free radical scavenging activity. Regular intake is linked to cardiovascular health, and their low toxicity and natural abundance support their potential as nutraceutical and pharmaceutical agents (Liu et al., 2024; Yao et al., 2004). Phenolic acids—such as caffeic, ferulic, and gallic acids—also possess strong antioxidant and anti-inflammatory effects through metal chelation and inhibition of pro-inflammatory enzymes like COX and LOX (Rashmi & Negi, 2020). A heatmap of significantly changed flavonoids and phenolic acids (*p* < 0.05) (Fig. 3d, e) revealed a general upregulation of flavones, flavanones, flavonols, and isoflavones, indicating that nano‑selenium broadly promoted flavonoid biosynthesis. Phenolic acid levels also trended upward, aligning with previous total flavonoid and phenol measurements. Notably, several vitamins were significantly upregulated by nano‑selenium, including l-ascorbic acid (Vitamin C), D-panthenol (Vitamin B5), dehydroascorbic acid (DHA), and pyridoxine-5’-O-glucoside (Vitamin B6). Only pyridoxine, another form of Vitamin B6, was downregulated. These changes suggest enhanced nutritional value of Leek moss due to increased levels of antioxidant compounds and essential vitamins. KEGG enrichment of the top 20 DAM-related pathways (Fig. 3g) highlighted significant enrichment in pathways such as biosynthesis of sinapic acid derivatives, quercetin aglycones, chrysoeriol aglycones, gentisic acid derivatives, and α-linolenic acid metabolism. Most, including chrysoeriol and quercetin aglycone biosynthesis, fall under flavonoid metabolism. Nano‑selenium's impact on α-linolenic acid metabolism has been reported in multiple species. The sinapic acid derivative biosynthesis pathway, a key branch of phenolic acid metabolism and lignin synthesis, may also influence Leek moss texture by altering lignin content.

### 3.4 The transcriptomic landscape of Leek moss under different forms of selenium treatments reveals unique effects of nano‑selenium

**Fig. 4:**
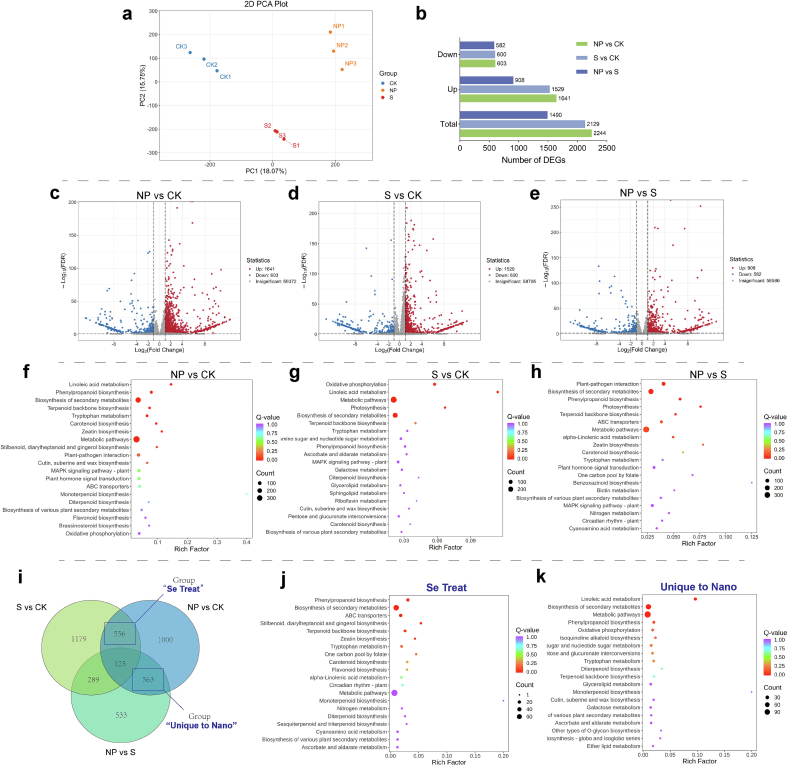
**Transcriptomic analysis.** (a) Principal component analysis (PCA); (b) Number of differentially expressed genes (DEGs), CK represents the control group, NP represents the nano-selenium treatment group (7.5 mg/L), and S represents the sodium selenite treatment group (7.5 mg/L).; (c–e) Volcano plots of DEGs between treatment groups; (f–h) KEGG pathway enrichment bubble plots of DEGs in each treatment group; (i) Venn diagram of DEGs among the three experimental groups; (j) KEGG enrichment bubble plot of DEGs between NP and CK in the “Se Treat” group, highlighting shared responses to different Se forms; (k) KEGG enrichment bubble plot of DEGs between NP and CK in the “Unique to Nano” group, highlighting nano-selenium-specific gene expression changes.

To further elucidate the biological mechanisms underlying nano‑selenium effects in *Allium tuberosum* (Leek moss), transcriptomic analysis was performed on control (CK), sodium selenite (S, 7.5 mg/L), and nano‑selenium (NP, 7.5 mg/L) groups. PCA (Fig. 4a) revealed clear transcriptomic separation among CK, S, and NP. A total of 4225 differentially expressed genes (DEGs) were identified across all comparisons, with selenium application generally inducing more gene upregulation. NP vs CK exhibited the highest number of DEGs, while NP vs S had the fewest (Fig. 4b), suggesting a more complex regulatory role of nano‑selenium than selenite. Specifically, 2244 DEGs (1641 upregulated, 603 downregulated) were found in NP vs CK (Fig. 4c); 2129 DEGs (1529 upregulated, 600 downregulated) in S vs CK (Fig. 4d); and 1490 DEGs (908 upregulated, 582 downregulated) in NP vs S (Fig. 4e). KEGG enrichment analysis revealed both shared and unique responses to different selenium forms. In NP vs CK (Fig. 4f), the top enriched pathways included linoleic acid metabolism, phenylpropanoid biosynthesis, secondary metabolite biosynthesis, terpenoid backbone biosynthesis, and tryptophan metabolism—largely consistent with metabolomic findings. In S vs CK (Fig. 4g), pathways such as oxidative phosphorylation, linoleic acid metabolism, photosynthesis, and secondary metabolite biosynthesis were enriched, showing overlap yet clear differences from the NP response. In NP vs S (Fig. 4h), DEGs were primarily enriched in plant-pathogen interaction, secondary metabolite biosynthesis, phenylpropanoid biosynthesis, photosynthesis, and terpenoid backbone biosynthesis. Notably, three of these are directly linked to secondary metabolism, supporting the notion that nano‑selenium more effectively stimulates secondary metabolite biosynthesis than selenite at the same concentration.

**Supplementary Figure S2:**
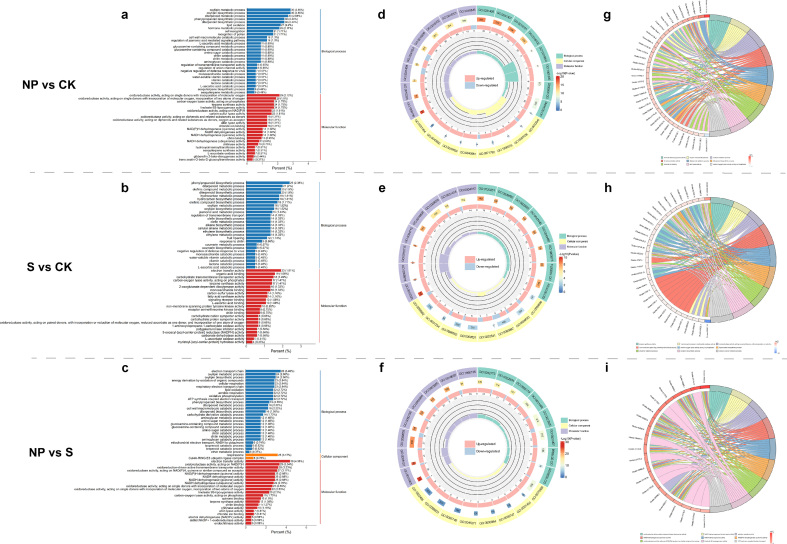
**GO functional enrichment analysis of differentially expressed genes.** (a–c) Bar plots showing the top 50 GO terms with the lowest q-values in each comparison group. (d–f) GO circle plots for each group, displaying the top 8 GO terms with the lowest q-values across the three GO categories—Biological Process, Cellular Component, and Molecular Function; if fewer than 8 terms were enriched, all are shown. (g–i) GO chord plots for each group, illustrating the top 9 GO terms with the lowest q-values and their associated genes. Each plot links the 10 DEGs with the highest |log₂FC| (left) to the most significantly enriched GO terms (right); connecting lines represent gene-term associations. The heatmap on the lower right indicates gene expression changes (log₂FC), with red denoting upregulated genes, blue downregulated genes, and color intensity reflecting the magnitude of differential expression.

A Venn diagram illustrated DEG distribution across comparisons (Fig. 4i). A total of 556 DEGs were shared by NP vs CK and S vs CK but not by NP vs S, representing a selenium-induced common response (“Se Treat” group, Fig. 4j). These genes were enriched in phenylpropanoid biosynthesis, secondary metabolite biosynthesis, and ABC transporters. The enrichment of ABC transporters—key mediators of selenium transport in plants (Xiao et al., 2024)—further supports a shared selenium-induced regulatory mechanism. In contrast, 563 DEGs were unique to NP vs CK and NP vs S, but absent in S vs CK, representing nano-specific responses (“Unique to Nano” group, Fig. 4k). In this group, the top three enriched pathways were linoleic acid metabolism, secondary metabolite biosynthesis, and phenylpropanoid biosynthesis, indicating that nano‑selenium exerts additional stimulatory effects on these pathways not observed with selenite, likely due to its enhanced physicochemical properties. GO enrichment analysis revealed functional characteristics of DEGs across groups (Fig. S2a-c), with results visualized using chord plots (Fig. S2d-f) and line plots (Fig. S2g-i). Compared with S, NP treatment led to greater enrichment in pathways related to reactive oxygen species (ROS) response and detoxification, consistent with nano‑selenium's superior antioxidant capacity observed in prior assays.

**Fig. 5:**
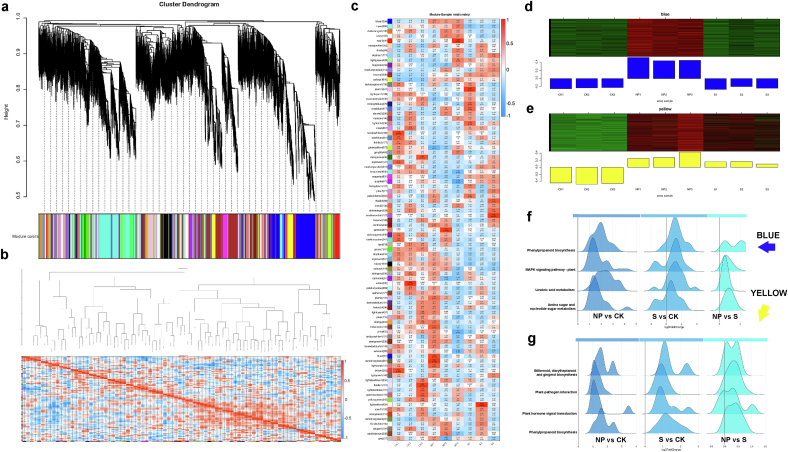
**WGCNA analysis.** (a) Hierarchical clustering of modules (merge threshold = 0.25; minimum module size = 50 genes), the upper part shows the gene dendrogram, while the lower part displays module clustering results, with each color representing a distinct module; (b) Sample correlation heatmap, the upper portion shows module clustering based on eigengene similarity, where the y-axis indicates node dissimilarity. In the lower heatmap, each row and column represent one module, and color intensity reflects the strength of correlation—darker (red) colors indicate stronger correlations, whereas lighter colors indicate weaker ones.; (c) Module–trait correlation heatmap, color intensity indicates the degree of correlation, where red denotes positive and blue denotes negative correlation; (d) Expression pattern of genes in the “blue” module; (e) Expression pattern of genes in the “yellow” module; (f-g) Distribution of “blue” and “yellow” module genes in significantly enriched pathways (NP vs CK), the y-axis shows the top four most significantly enriched pathways, and the x-axis represents the distribution of genes in these pathways according to their log₂FC values—the higher the peak, the greater the number of genes within that log₂FC range. (For interpretation of the references to color in this figure legend, the reader is referred to the web version of this article.)

Beyond differential gene expression, expression patterns in response to external stimuli provide key insights into regulatory mechanisms. To explore the relationship between gene expression modules and their potential biological significance following selenium application, we performed Weighted Gene Co-expression Network Analysis (WGCNA) based on the transcriptomic data. A hierarchical clustering tree was constructed using pairwise gene expression correlations, with a module merging threshold of 0.25 and a minimum module size of 50 genes, resulting in 87 distinct co-expression modules (Fig. 5a). Module dissimilarity was assessed through a correlation heatmap to confirm their uniqueness (Fig. 5b). The correlation heatmap between modules and treatment samples (Fig. 5c) revealed that the “blue” and “yellow” modules corresponded closely with the previously defined “Unique to Nano” and “Se Treat” groups, respectively. The “blue” module contained 4259 genes, all upregulated specifically under nano‑selenium treatment but not in CK or sodium selenite, reflecting a nano-specific expression pattern (Fig. 5d). The “yellow” module included 1816 genes that were generally upregulated in response to selenium, with a stronger response under nano‑selenium, consistent with physiological and biochemical trends (Fig. 5e). KEGG enrichment analysis of both modules, visualized via ridge plots, highlighted distinct pathway distributions and biological functions. In the “blue” module, genes were primarily enriched in phenylpropanoid biosynthesis, MAPK signaling pathway – plant, linoleic acid metabolism, and amino sugar and nucleotide sugar metabolism (Fig. 5f). Notably, the expression patterns in NP vs CK were markedly distinct from the other comparisons. This enrichment profile reinforces previous DEG-based findings for the “Unique to Nano” group, where MAPK signaling and amino sugar metabolism were prominent, and phenylpropanoid biosynthesis remained the most significantly enriched pathway—underscoring nano‑selenium's specific activation of phenylpropanoid metabolism. In the “yellow” module, in addition to phenylpropanoid biosynthesis, pathways such as stilbenoid, diarylheptanoid and gingerol biosynthesis, plant-pathogen interaction, and plant hormone signal transduction were also significantly enriched (Fig. 5g). These results suggest that selenium application not only enhances secondary metabolism but may also influence plant defense mechanisms and hormonal regulation.

### 3.5 Nano‑selenium reduces spiciness and improves texture of Leek moss

**Fig. 6:**
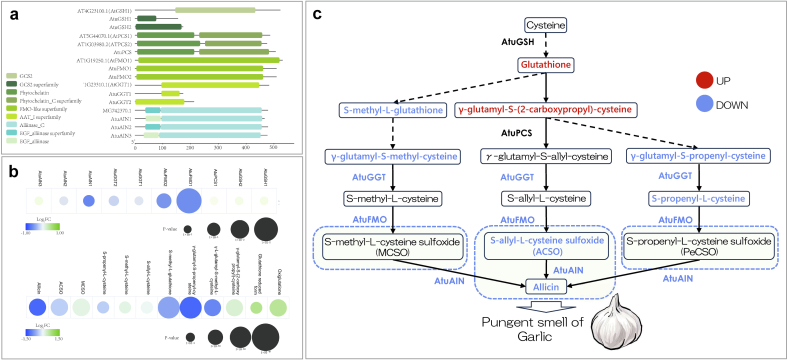
**Effects of Nano-seleniumlenium on Flavor Compound Metabolism in Chinese Chive.** (a) Domain architecture of key enzymes and reference genes involved in the CSO (cysteine sulfoxide) metabolic pathway in Chinese chive; (b) Bubble plot showing relative abundance of CSO-related metabolites and expression levels of corresponding enzyme genes; (c) Proposed mechanism by which nano-selenium modulates flavor compound metabolism in Chinese chive.

To investigate the effect of nano‑selenium on the flavor of Leek moss, we mapped key genes and metabolites to the CSO (cysteine sulfoxide) biosynthesis pathway based on previous studies (Li et al., 2025; Liu, Tong, Hu, Ji, & Wu, 2021). Due to the lack of a reference genome, CSO-related genes were identified from transcriptomic data using homologs reported in model species or the same genus. Candidate genes were further validated through Pfam domain analysis against reference proteins (Fig. 6a), resulting in the identification of 10 CSO biosynthesis-related enzyme genes. From metabolomic profiling, 12 intermediates were identified, and their accumulation, along with gene expression patterns, was visualized via a bubble plot (Fig. 6b). Key intermediates—*S*-methyl-L-glutathione, γ-glutamyl-*S*-methyl-cysteine, γ-glutamyl-S-propenyl-cysteine, and S-propenyl-cysteine—were significantly downregulated. The contents of ACSO and the final product allicin were reduced to 81.8% and 27.4% of CK levels, respectively. Correspondingly, the expression of key biosynthetic enzymes—including *AtuGGT*, *AtuFMO*, and *AtuAIN* involved in allicin formation—was markedly suppressed. Specifically, Log₂FC values for *AtuGGT1* and *AtuGGT2* were −0.21 and −0.40; for *AtuFMO1* and *AtuFMO2*, −1.10 and −1.01; and for *AtuAIN1*, *AtuAIN2*, and *AtuAIN3*, −1.17, −0.23, and 0.16, respectively. Interestingly, ACSO was found to be the dominant sulfoxide, in contrast to prior reports identifying MCSO as the major component in Allium species (Liu et al., 2022). This discrepancy may result from tissue specificity or varietal differences, as earlier studies focused on leaves, whereas our analysis targeted the flowering stalk. In summary, multi-omics integration revealed that nano‑selenium suppresses the biosynthesis of pungent CSO-derived compounds, leading to reduced allicin-associated garlic-like flavor (Fig. 6c). As consumer preferences for aroma and taste vary, it remains inconclusive whether the diminished pungency enhances or diminishes overall flavor perception.

**Fig. 7:**
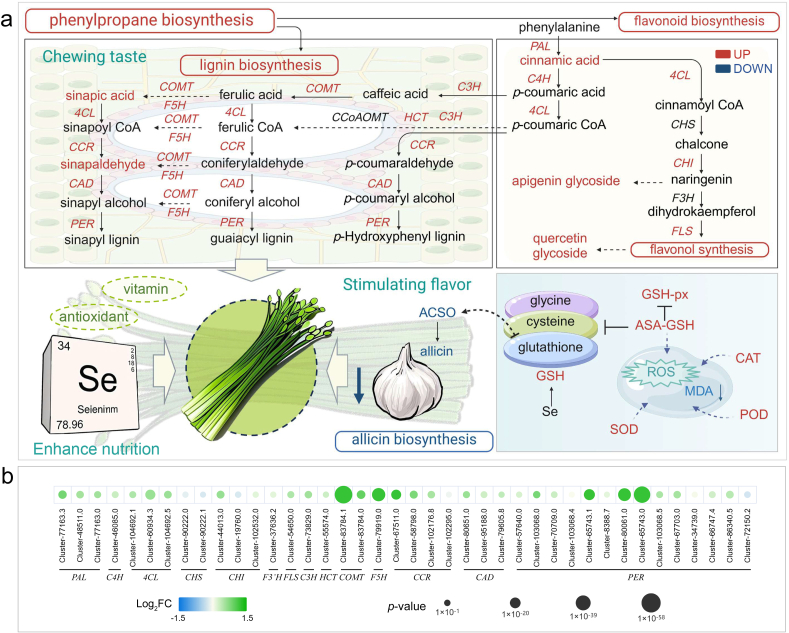
**Simplified Regulatory Mechanism of Exogenous Nano-selenium in Chinese Chive.** (a) Schematic diagram of the proposed regulatory mechanism; (b) Expression levels of DEGs involved in the mechanism.

Beyond flavor, texture is a key determinant of a food ingredient's value. Compared to Leek leaves, Leek moss offers a more complex mouthfeel, particularly a crisp texture upon chewing. Lignin accumulation in plant cell walls significantly enhances tissue rigidity and toughness, thereby reducing chewability and altering texture. As lignin levels increase, plant tissues exhibit greater resistance to deformation and require higher shear force during mastication, resulting in a firmer, more brittle texture that is harder to chew (Wang et al., 2023). Lignin biosynthesis is a major branch of the phenylpropanoid pathway. Our integrated multi-omics analysis revealed that nano‑selenium not only activated phenylpropanoid metabolism but also promoted lignin biosynthesis in Leek moss (Fig. 7a). Key lignin biosynthetic enzyme genes—including CCR, CAD, and PER—were significantly upregulated, along with upstream genes C3H and HCT, and precursor-modifying genes COMT and F5H (Fig. 7b). This regulation was associated with increased sinapic acid levels, a critical phenylpropanoid intermediate. Sinapic acid is converted to sinapoyl-CoA and subsequently to sinapyl alcohol, which polymerizes into syringyl lignin (Yamauchi, Yasuda, & Fukushima, 2002). In our study, sinapic acid levels increased 2.12-fold following nano‑selenium treatment, accompanied by strong upregulation of the downstream genes involved in S-lignin synthesis, indicating enhanced syringyl lignin accumulation. In summary, Fig. 7 highlights the comprehensive effects of nano‑selenium on Leek moss: it enhances nutritional quality and antioxidant capacity, reduces pungent flavor, and improves texture by promoting lignin biosynthesis, ultimately contributing to a crisper and more palatable eating experience.

## 4 Discussion

In this study, field experiments demonstrated that nano‑selenium markedly enhanced secondary metabolism and antioxidant capacity in leek moss, while also modulating its sensory and edible qualities. Nano‑selenium treatment significantly increased the activities of antioxidant enzymes (SOD, POD, CAT, and GSH-Px) as well as the levels of small-molecule antioxidants such as GSH. Metabolomic profiling revealed a reduction in flavor-related organosulfur compounds, accompanied by a notable accumulation of health-promoting metabolites, including phenylalanine derivatives and flavonoids. These findings are consistent with Chen et al. (Chen, Zhang, Peng, Zhu, & Wang, 2023), who reported elevated AsA–GSH cycle components and phenolic compounds in Chinese broccoli following selenite application. Importantly, the persistence of field effects was compared between nano‑selenium and inorganic sodium selenite. Nano‑selenium maintained its metabolic enhancement for at least three weeks, whereas selenite exhibited a shorter duration and weaker magnitude of response—a key insight for crops harvested continuously. Concentration-gradient experiments further revealed that nano‑selenium achieved optimal efficacy at 7.5–10 mg/L, while selenite showed no distinct optimal range within 0–15 mg/L.

Selenium and sulfur belong to the same chalcogen group and exhibit comparable chemical behaviors. In plants, they share overlapping uptake and metabolic pathways and frequently compete during absorption and assimilation (Ji et al., 2025). Such Se—S competition can influence the biosynthesis of sulfur-containing volatiles, thereby modifying the flavor profile of Allium species. Our results demonstrated that nano‑selenium treatment decreased the accumulation of sulfur volatiles in leek moss, resulting in a noticeably milder pungency. Similarly, Ogra et al. reported that exogenous selenium application reduced the characteristic sharpness of selenium-enriched garlic and scallions, attributing this effect to the depletion of key sulfurous precursors in Allium crops (Ogra, Ishiwata, Iwashita, & Suzuki, 2005). Kopsell and Randle also observed that higher selenium application levels diminished onion pungency without inhibiting sulfur uptake, implying that selenium disrupts the metabolism of sulfur-based flavor compounds (Kopsell & Randle, 1997). Multi-omics analysis further revealed a reduction in sulfur-containing metabolites such as alliin, accompanied by the downregulation of key catalytic genes (AtuGGT, AtuFMO, and AtuAIN). This phenomenon likely results from Se—S competition at sulfate transport sites, where selenium incorporation interferes with the activity of sulfur-metabolizing enzymes, thereby triggering feedback inhibition of related gene expression. Future studies using isotopic labeling and tracing approaches could help clarify the exact mechanisms through which Se—S competition modulates CSO metabolic pathways under nano‑selenium treatment.

This study compared gene expression responses in Leek moss under equal concentrations (7.5 mg/L) of nano‑selenium and sodium selenite treatments. Notably, NP significantly enriched secondary metabolism pathways such as linoleic acid metabolism and phenylpropanoid biosynthesis compared to the control. Additionally, disease resistance-related pathways like plant–pathogen interaction were specifically enriched in the NP vs. S comparison. WGCNA identified a “blue” module specifically associated with NP treatment, enriched in signal transduction pathways including MAPK signaling. These findings suggest that NP more strongly activates defense-related pathways, including phenylpropanoid metabolism, reactive oxygen species (ROS) responses, and signal transduction, compared to inorganic Se. The distinct bioactivity of nano‑selenium likely arises from its nanoscale properties. Nanoparticles offer higher surface area-to-volume ratios and controlled release kinetics, improving nutrient delivery and prolonging biological effects. Samynathan et al. highlighted that nanoparticles not only enhance elemental uptake via controlled release and targeted delivery but also modulate plant gene expression directly (Samynathan et al., 2023). Hu et al. further reported that 40 nm nano‑selenium was absorbed by wheat roots at 1.8 times the efficiency of 140 nm and 240 nm particles, indicating enhanced bioavailability at smaller sizes (Hu et al., 2018). In summary, transcriptomic and metabolomic analyses confirmed that nano‑selenium activated phenylpropanoid and MAPK signaling pathways, promoting lignin and flavonoid biosynthesis while suppressing sulfur-containing volatiles. These integrated effects enhance both antioxidant capacity and sensory quality, positioning nano‑selenium as a promising biofortification agent for sustainable vegetable production. However, challenges remain regarding the long-term environmental impact, bioaccumulation potential, and stability of nanomaterials under diverse field conditions. Future research should focus on optimizing nano‑selenium dosage, understanding its interactions with plant-soil-microbe systems, and developing scalable, eco-safe application strategies for agricultural use.

## CRediT authorship contribution statement

**Mufan Hu:** Writing – original draft, Visualization, Methodology, Formal analysis. **Hongmei Wang:** Writing – original draft, Investigation, Formal analysis. **Cuina Fu:** Visualization, Validation, Resources. **Kouhua Yu:** Methodology, Formal analysis. **Fengqiong Liu:** Validation, Investigation, Funding acquisition. **Qingwei Tan:** Visualization. **Yijie Li:** Resources, Investigation. **Qinyong Dong:** Visualization. **Feng Zhang:** Validation. **Shenkui He:** Resources. **Jingcheng Wu:** Methodology. **Gengsheng Mo:** Resources. **Yuanhui Xiao:** Writing – review & editing, Funding acquisition, Formal analysis. **Canping Pan:** Writing – review & editing, Supervision, Resources, Formal analysis, Conceptualization.

## Declaration of competing interest

The authors declare that they have no known competing financial interests or personal relationships that could have appeared to influence the work reported in this paper.

Acknowledges.

The work is funded by Guangxi Key Technologies R&D Program, AB25069395 and the 2115 Talent Development Program of China Agricultural University.

## Data Availability

Data will be made available on request.
